# Production of polyhydroxyalkanoates from waste frying oil by *Cupriavidus necator*

**DOI:** 10.1186/2191-0855-1-11

**Published:** 2011-06-10

**Authors:** Rob AJ Verlinden, David J Hill, Melvin A Kenward, Craig D Williams, Zofia Piotrowska-Seget, Iza K Radecka

**Affiliations:** 1 University of Wolverhampton, School of Applied Sciences, Wulfruna Street, WV1 1SB, Wolverhampton, UK; 2 University of Silesia, Department of Microbiology, Jagiellonska 28, 40-032 Katowice, Poland

**Keywords:** polyhydroxyalkanoates, polyhydroxybutyrate, bacterial fermentation, biopolymer, waste frying oil, rapeseed oil

## Abstract

Polyhydroxyalkanoates (PHAs) are biopolymers, which can replace petrochemical plastics in many applications. However, these bioplastics are currently far more expensive than petrochemical plastics. Many researchers are investigating the use of inexpensive substrates derived from waste streams. Waste frying oil is abundant and can be used in PHA production without filtration.

*Cupriavidus necator *(formerly known as *Ralstonia eutropha*) is a versatile organism for the production of PHAs. Small-scale batch fermentation studies have been set up, using different concentrations of pure vegetable oil, heated vegetable oil and waste frying oil. These oils are all rapeseed oils.

It has been shown that *Cupriavidus necator *produced the homopolymer polyhydroxybutyrate (PHB) from the rapeseed oils. The achieved PHB concentration from waste frying oil was 1.2 g/l, which is similar to a concentration that can be obtained from glucose. The PHB harvest from pure oil and heated oil was 0.62 g/l and 0.9 g/l respectively. A feed of waste frying oil could thus achieve more biopolymer than pure vegetable oil. While the use of a waste product is beneficial from a life-cycle perspective, PHB is not the only product that can be made from waste oil. The collection of waste frying oil is becoming more widespread, making waste oil a good alternative to purified oil or glucose for PHB production.

## Introduction

Polyhydroxyalkanoates (PHAs) are natural, renewable and biocompatible biopolymers, produced intracellular in bacteria. They can be made into plastic materials with properties that are similar to petrochemical plastics and can replace these materials in many applications (Philip et al. 2007). However, the high production cost of biopolymers and the availability of low-cost petrochemical equivalents make polyhydroxyalkanoates economically unattractive. Concern over plastic waste and increasing environmental awareness has put bioplastics into the attention of research and industry. In order to make the production of PHAs economically more attractive, the use of inexpensive substrates has been investigated thoroughly (Lemos et al. 2006; Castilho et al. 2009).

Vegetable oils have been found to be possible substrates in the production of PHAs (Alias and Tan 2005; Kahar et al. 2004). Waste streams from oil mills or used oils, which are even cheaper than purified oils can be used too (Fernández et al. 2005; Mumtaz et al. 2010).

The study described in this paper focussed on the use of waste frying oil for the production of PHAs. Since deep frying is popular, there is a potential to turn this waste resource into a useful biomaterial. It was chosen to use rapeseed oils, since it is a common frying oil in Europe. In other regions of the world the nature of frying oil can differ. Waste rapeseed oil has been used successfully for the production of PHAs with valerate monomers (Obruca et al. 2010). Waste frying oil has to be filtered for the production of soap or biodiesel, but it can be used in PHA-production without filtration.

Out of the many different bacterial cultures that can produce PHAs (Lee 1996), *Cupriavidus necator *has been most extensively studied (Volova and Kalacheva 2005; Yan et al. 2005). At present, bacterial fermentation of *Cupriavidus necator *is used widely in industrial processes towards PHAs (Khanna and Srivastava 2005a). Wild-type *C. necator *is known as a poor oil utiliser, but studies into novel plant oil media can increase this yield (Budde et al. 2011).

In this study the production of PHAs with *Cupriavidus necator *from waste and heated frying oil was studied in two bacterial growth media and compared to the production of these biopolymers from pure vegetable oil.

## Materials and methods

### Chemicals

K_2_HPO_4 _and KH_2_PO_4 _were obtained from Fisher Chemicals Ltd., Loughborough, UK. Chloroform (HPLC grade), n-hexane and methanol were obtained from Rathburn Chemicals Ltd., Walkerburn, UK. Sulphuric acid was purchased from Scientific & Chemical Supplies Ltd., Bilston, UK. Methyl benzoate, KNO_3_, (NH_4_)_2_SO_4_, MgSO_4_.7H_2_O, NaCl, CuSO_4_.5H_2_O, MnSO_4_.5H_2_O, ZnSO_4_.5H_2_O, CaCl_2_, FeSO_4 _and (NH_4_)_6_Mo_7_O_24_ .4H_2_O were purchased from BDH Chemicals Ltd., Poole, UK.

### Vegetable oils

Pure vegetable oil, from rapeseed, was obtained from a local supermarket. Waste frying oil was provided by a food establishment, which deep-fries chips and chicken. Heated oil was obtained by heating pure vegetable oil in an open container at 180°C for 1 week. 180°C is a common frying temperature (Costa et al. 2001). All oils were sterilised separately in a steam autoclave and added directly to fermentations. Since the oils were either refined or subjected to prolonged heating, only few spores were present in the oils. Speadplating of 1 ml of oil on TSA showed no colonies after incubation at 30°C for 24 hours.

### Growth media

Tryptone Soya Broth (TSB) contains distilled water, 17 g/l Tryptone, 3 g/l Soy Peptone, 5 gl/l NaCl, 2.5 g/l K_2_HPO_4 _and 2.5 g/l D-glucose.

Tryptone Soya Agar (TSA) contains distilled water, 15 g/l Tryptone, 5 g/l Soy Peptone, 5 g/l NaCl and 12 g/l Agar No. 2.

Basal Salts Medium (BSM) contains distilled water, 1 g/l K_2_HPO_4_, 1 g/l KH_2_PO_4_, 1 g/l KNO_3_, 1 g/l (NH_4_)_2_SO_4_, 0.1 g/l MgSO_4_.7H_2_O, 0.1 g/l NaCl, 10 ml/l Trace elements solution. Trace element solution has: 2 mg/l CaCl_2_, 2 mg/l CuSO_4_.5H_2_O, 2 mg/l MnSO_4_.5H_2_O, 2 mg/l ZnSO_4_.5H_2_O, 2 mg/l FeSO_4_, 2 mg/l (NH_4_)_6_Mo_7_O_24_.4H_2_O.

Basal Salts Agar (BSA) contains all ingredients of BSM and additionally 15 g/l Agar No. 2.

#### Nitrogen Concentration in Media

It has been reported that the nitrogen concentration in bacteriological media highly influences the production of intracellular PHAs (Khanna and Srivastava 2005b). From EDX (Energy Dispersive X-Ray) experiments it was found that TSB contains around 4.4 g/l of nitrogen. Most of this is probably available from short amino acids. From the chemical formulas of its constituents it was calculated that BSM contained approximately 0.35 g/l of nitrogen. The main nitrogen sources in BSM are nitrate and ammonium.

### Culture conditions

*Cupriavidus necator *H16 (NCIMB 10442, ATCC 17699) was obtained from NCIMB, Aberdeen, United Kingdom, and subcultured on Tryptone Soya Agar (TSA).

25 ml of Tryptone Soya Broth (TSB) was inoculated from a single colony and incubated for approximately 24 hours at 30°C. Cultures were checked for purity by Gram staining and observed under a microscope at 1000 ×.

Batch fermentations were performed in Erlenmeyer flasks of 500 ml. The flasks were filled with 250 ml of medium, including the 25 ml TSB inoculum, resulting in an inoculation ratio of 10% (v/v). The initial viable cell number was 2.10^7^-1.10^8 ^cfu/ml. Either a quantity of pure vegetable oil, heated vegetable oil or waste frying oil was added to the fermentation medium. The initial oil concentrations were 20 g/l. After addition of the oil, but before inoculation, the sterile medium was sonicated for 10 minutes to achieve a homogenised mixture.

All flasks were incubated in a rotary incubator (150 rpm) at 30°C and fermentations were stopped after certain times. All experiments were done in triplicate. The fermentation broth was centrifuged at 2500 × g for 10 minutes. The bacterial pellet was lyophilised and the supernatant kept for analysis.

### Viable Cell Count and Total Cell Dry Weight

After ten-fold serial dilutions the number of viable cells was assessed by spread plating of 100 μl on TSA plates. To determine the total cell dry weight, a known volume of the fermentation broth was centrifuged at 2500 × g for 10 minutes and the supernatant was separated off. The bacterial pellet was lyophilised for 24 hours and weighed.

### Determination of PHB content by Gas Chromatography

The amount of PHB in the bacterial biomass was determined using the method developed by Braunegg et al (1978). Additions made to the original method by Jan et al. (1994) and Yunji Xu (Koutinas et al. 2007) were implemented.

Freeze-dried biomass was grinded to a powder and put into a gas-tight screw-capped tube. 2 ml acidified methanol (3% v/v sulphuric acid, 2.5 g/l methyl-benzoate) and 2 ml chloroform were added. The methyl-benzoate was used as an internal standard to improve accuracy. The tube was closed and kept at 90-100°C for 3 hours. After rapid cooling, 4 ml of distilled water was added to achieve a good phase separation. The sample was vortexed for 10 seconds and the chloroform-phase was then filtered through a PTFE-filter and 1 μl was injected into a gas chromatograph (GC). The used GC was fabricated by the Thermo Finnigan Corporation, Milan, Italy. It had a Restek capillary column (Rtx-5MS) 30 m × 0.25 mm × 0.25 μm. Gas flow: 1.5 ml/ min. Carrier gas: He and make-up gas: N_2_. Column temperature range: 100-160°C. Temperature gradient: 15°C/ min. Inlet temperature: 250°C. Split ratio: 40. Detector temperature: 300°C. Detector type: flame ionisation.

A calibration curve was constructed using pure PHB from solvent extraction. The PHB content is determined from the peak-areas of the methyl-3-hydroxybutyrate and the internal standard.

### Solvent extraction

Up to 5 grams of lyophilised biomass was transferred into an extraction thimble. In a soxhlet extractor the PHAs ware extracted with approximately 250 ml of chloroform during 3 hours.

The hot solution of polymer was concentrated by evaporation. Subsequently the solution was precipitated in n-hexane (1:4, v:v) with a dropping pipette for purification. The polymer precipitated as a white substance.

### Fatty acid analysis

1 mg of pure oil and 1 mg of waste oil were added to separate clean glass tubes. 1 ml of saponification reagent was added, consisting of a 150 g/l NaOH-solution in methanol/distilled water 1:1 (v:v). The tubes were closed with a PTFE-lined cap and mixed on a vortex shaker for 10 seconds. The tubes were then placed in a water bath at 100°C for 30 minutes.

After cooling, 2 ml of methylation agent was added. The methylation agent was a 13:11 (v:v) mixture of 6.00 N hydrochloric acid and methanol. The tubes were vortexed and kept at 80°C for 10 minutes.

Again the tubes were cooled to room temperature and 1.25 ml of extraction solvent (a solvent with even volumes of hexane and methyl tert-butyl ether) was added. For 10 minutes the tubes were then mixed slowly, end-over-end, to achieve a good mass transfer to the solvent. The organic (top) phase was washed with a sodium hydroxyl solution and injected in a gas chomatograph (GC). The used GC had a 25 m × 0.2 mm capillary column and a temperature trajectory of 170-270°C was used at a rate of 5°C/min. Gas flow: 1.5 ml/ min. Carrier gas: He and make-up gas: N_2_. Detector type: flame ionisation. Fatty acids were identified with the Sherlock identification system.

### Nuclear Magnetic Resonance (NMR)

Samples were dissolved in deuterated chloroform (CDCl_3_) and ^1^H NMR spectra were recorded on a Bruker AV-400 NMR spectrometer at 400 MHz.

### Size Exclusion Chromatography (SEC)

Size exclusion chromatography (SEC) was used to assess the molecular weight distribution of polymers in solution based on a standard curve (Wong et al. 2004). The SEC system consists of a Polymers Labs (UK) pump connected to a PLgel, 10 μm, MIXED column. Chloroform (HPLC grade) was used as the eluent at 1 ml/min. Before use the column was calibrated with polystyrene standards of molecular weights between 580 and 3,000,000 Da.

### PHB concentration and statistical analysis

In one-stage batch fermentations bacterial cells grow in cell number and mass and simultaneously accumulate intracellular polymer. The PHB concentration, which is found by multiplying the total cell dry weight (g/l) by the PHB content (wt%), is the quantity that gives an indication when the broth contains an optimal amount of PHB. Fermentation data were fitted with second-order polynomial functions. Two-way ANOVA was used to compare data sets in GraphPad Prism. The highest PHB data points were compared to the next lower ones with a Student t-test.

## Results

### Fermentations

**Figure 1:**
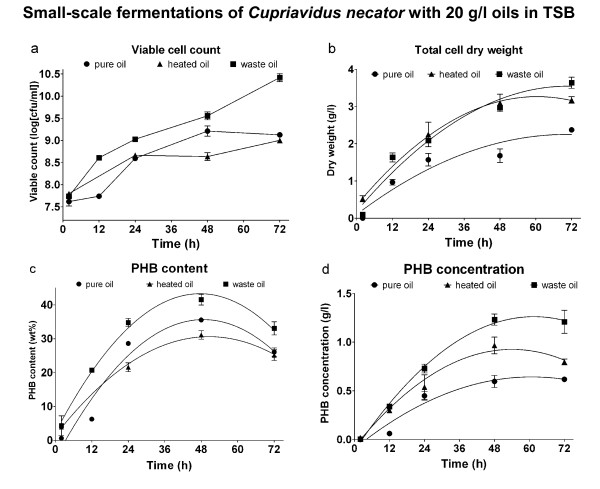
**Viable cell count (a), total cell dry weight (b), PHB content (c) and PHB concentration (d) of fermentations with a starting concentration of 20 g/l oils in TSB**. Data points are arithmetic means of triplicates, while error bars denote the standard error of the mean.

The results from small-scale fermentations with 20 g/l oils in Tryptone Soya Broth (TSB) are shown in Figure 1. As seen in Figure 1a, the initial viable cell number was similar for pure, heated and waste oil (about 4.10^7 ^cfu/ml). After 72 hours the viable cell count for the waste-oil fermentation had risen to 3.10^10^, while the other two fermentations only had 1.10^9 ^cfu/ml of viable cells. While there were more viable cells in the fermentations with waste oil, the total cell dry weight for heated oil and pure oil were found to be similar (Figure 1b). The PHB-content for all three fermentations have and optimum around 48 hours. PHB levels are decreasing after this time, due to utilisation of PHB as a carbon source in the metabolism (Figure 1c). The resulting curves for the PHB concentrations in fermentations with oils are found to be distinctly different (Figure 1d). While the most PHB is produced in a fermentation with waste oil (1.2 g/l was achieved after 72 hours), only 0.62 g/l was synthesised after 72 hours using pure vegetable oil as a carbon source. A fermentation with heated oil achieved a PHB concentration of 0.9 g/l after 48 hours, while the concentration was 0.8 g/l after 72 hours. Analysing the data for the PHB concentration, ANOVA shows that waste oil performs significantly better than pure oil from 12 hours onwards (P < 0.01). While the data points for heated oil all lie above those for pure oil, there is only significant difference in the t-test at 48 hours (Figure 1d).

**Figure 2:**
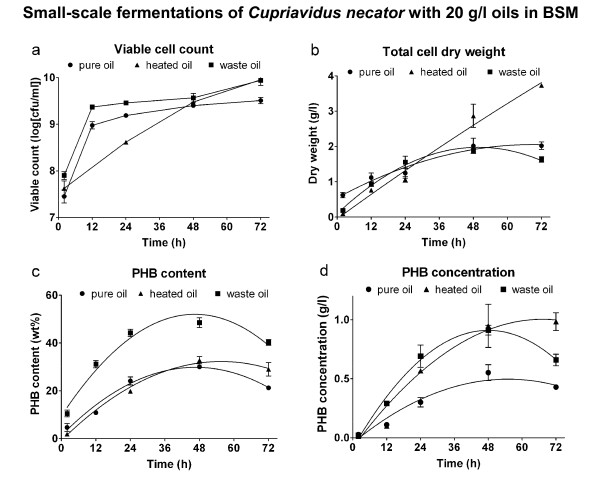
**Viable cell count (a), total cell dry weight (b), PHB content (c) and PHB concentration (d) of fermentations with a starting concentration of 20 g/l oils in BSM**. Data points are arithmetic means of triplicates, while error bars denote the standard error of the mean.

The results from fermentations of *Cupriavidus necator *with 20 g/l oils in Basal Salts Medium (BSM) are shown in Figure 2. It can be seen that the initial viable cell number (Figure 2a) of all fermentations were around 5.10^7^, which increased to around 4.10^9 ^in 72 hours. The total cell dry weight of a heated-oil fermentation in BSM was substantially higher at 48 and 72 hours than pure and waste oils (3.7 g/l compared to 2 g/l in Figure 2b).

The PHB-content curve of heated oil was similar to the curve of pure oil (Figure 2c), while the waste-oil curve was higher for all fermentation times. Looking at the PHB concentration of heated oil and waste oil, they were both above pure oil (Figure 2d). From ANOVA it was concluded that waste and heated oil yield a better PHB concentration than pure oil for times longer than or equal to 24 hours (P < 0.05). In the medium with waste oil the PHB concentration decreased from 0.95 to 0.65 g/l after 48 hours, while the heated-oil curve continued to rise to about 1.0 g/l. The PHB concentration achieved with pure oil was 0.55 g/l after 48 hours and 0.42 g/l after 72 hours.

Fermentations of *Cupriavidus necator *with waste frying oil obtained a significantly higher PHB concentration than with pure vegetable oil. While a maximum concentration of 1.2 g/l can be achieved with waste oil, only 0.62 g/l was synthesised using pure oil as a carbon source. The production of PHB was found to be better with waste oil in two growth media, which had different amounts of available nitrogen.

The improved performance of waste oil compared to pure oil could be ascribed to the prolonged heating of waste oil. Rapeseed oil that had been heated for around 1 week was also used in fermentations. The maximum PHB concentration with heated vegetable oil was 0.98 g/l in a fermentation using 20 g/l of the oil. Heated oil gave better results for PHB concentration than pure oil, although the improvement was less than from waste oil. Heating seemed to be a major factor in the improvement of PHB production from vegetable oils.

### Fatty acid analysis of vegetable oils

**Figure 3:**
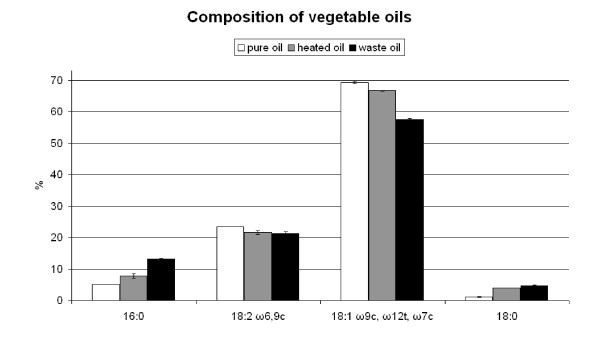
**The composition of pure vegetable oil (untreated), after 1 week of heat treatment (heated oil) and after deep-frying (waste oil)**. Data in the graph are arithmetic means of duplicates, while error bars denote the standard error of the mean.

The results of fatty acid analysis are shown in Figure 3. When comparing pure oil to heated oil, it can be seen that the amount of 16:0 fatty acids increased, while the amount of 18:2 decreased. The fraction of 18:1 ω9 c remained at approximately the same level, while after 2 weeks of heating a significant amount of 18:1 ω12 t/ω7 c could be observed. Thus, heating the oil changed the composition and reduced the total amount of unsaturations in the oil.

Figure 3 also shows that there are differences between the waste oil (black bars) the heated oils. Compositional differences can be due to the interaction with food and moisture with the waste frying oil, which would not occur in heated oil. The amount of 16:0 fatty acids present in waste oil is an indicator that it was heated for about 2 weeks.

### Polymer analysis

**Figure 4:**
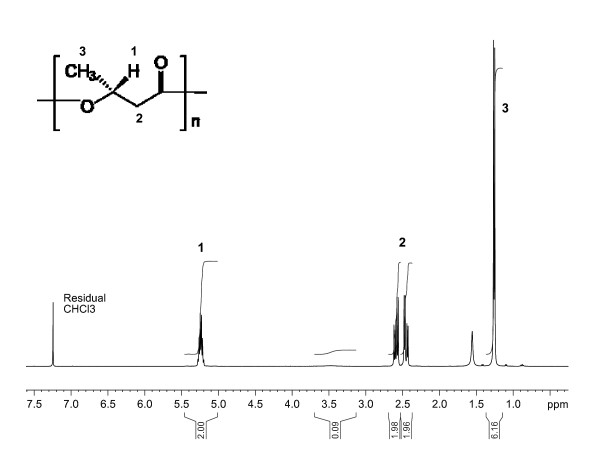
**^1^H NMR spectrum of PHB produced from waste frying oil**.

PHB from glucose and oils was analysed by SEC and found to have molecular weights (Mn) between 2.10^5 ^and 2.10^6 ^with an average polydispersity of around 3.5. The NMR spectra of the polymer from waste frying oil (Figure 4), pure vegetable oil and glucose indicated the sole presence of pure polyhydroxybutyrate (PHB) in all three cases.

## Discussion

### Fermentation productivity

The maximum production of PHB from waste frying oil (1.2 g/l) was achieved after 72 hours of fermentation in TSB. The PHB production results in this study were low in comparison to fermentations with *Cupriavidus necator *using various oils, performed by del Rocio et al. (2007). They achieved PHA concentrations between 3 to 6 g/l in 25 hours and used the medium and conditions as described by Yu et al. (2002). The medium contains both salts and extracted nutrient components, which may cause the higher production.

### Nitrogen content of media

The amount of available nitrogen in the media has a great influence on cell growth and PHB accumulation. More nitrogen has been shown to increase the growth and PHB production of *Cupriavidus necator *(Wang and Yu 2001; Wang et al. 2007). For all three substrates in this study the bacteria achieve higher dry cell mass and thus a higher concentration of PHB in TSB (high nitrogen) than in BSM (low nitrogen). In heated or waste oil there may be more nitrogen present than in the untreated pure oil, since thermal degradation products can contain readily available nitrogen.

### The composition of vegetable oils

Since fermentations produce more PHB when waste frying oil is used (compared to pure vegetable oil) the compositional changes during the frying process play an important role in the bacterial conversion to PHB. 98-99% of the components of pure oils are fatty acids (Tabee 2008). During frying complex components are formed in the oil and residue of food products are added to the oil. It is known that many chemical processes occur during the prolonged heating of vegetable oils under influence or air and moisture. The dominant process during heat treatment of oils is oxidation of fatty acids under influence of oxygen (Boskou and Elmadfa 1999).

To find out more about compositional changes of oils, fatty acid analysis was performed. The composition of fatty acids with a carbon length between 12 and 20 were detected with the Sherlock identification method. However, it is also known that heating causes products to be formed which can not be detected by the used Sherlock identification method. Examples are: peroxides, compounds with relatively short carbon chain length and polar compounds (Choe and Min 2007).

The results from the fatty acid analysis are indicating that saturated fatty acids lead to build-up of more energy-rich PHB in bacteria than with unsaturated fatty acids. Traditionally the PHB synthesis mechanism has been described as a *de novo *route in which fatty acids are transformed into acetyl-CoA by β-oxidation cycles (Rehm et al. 1998). It is possible that saturated fatty acids are more easily converted to acetyl-CoA than unsaturated fatty acids. However, in pure oil, heated oil and waste oil, there is an excess quantity of unsaturated fatty acids present. Benefits for the PHB production are likely to be found in the growth of the bacterial organisms instead of the PHB metabolism.

A full content analysis of pure oil, heated oil and frying oil should to be done to assess the components in the oils that improve growth and PHB accumulation. Compounds of interest in waste oils are foreign food residues, readily available nitrogen compounds, peroxides and short-chain compound formed during heating.

From fatty acid analysis it was shown that the amount of unsaturations in oils is reduced during heating and frying. Since the fatty acid composition of waste oil shows that there are still many unsaturated fatty acids present, the fatty acids composition may not be the only factor contributing to increased performance. Residual carbohydrates, proteins and fats from foods, available nitrogen compounds, peroxides and heat-degradation products could also be metabolised and may have contributed to increased PHB production.

### High quality PHB from a waste material

It has been proven that when *Cupriavidus necator *grows on a feed of alternative substrates, such as valerate, octanoate or oils (Volova and Kalacheva 2005) copolymers were formed. However, when grown on oils the biopolymer produced was found to be chemically pure PHB from pure oil, heated oil and waste oil. The molecular weights of polymers from waste frying were similar to those from other oils and glucose. Thus producing PHB from waste frying oil does not impact on the molecular properties of the final polymer.

For the PHA production with *Cupriavidus necator *waste rapeseed oil is a good option. While Obruca et al. (2010) prove efficient production of co-polymers from waste oil, this study compared waste oil to pure oil and also to glucose. The PHB concentration achieved from waste oil was in TSB (1.2 g/l) was similar to a fermentation with 20 g/l glucose in TSB (unpublished data). When replacing purified glucose as a carbon source, waste oils yield a similar amount of PHB.

As a practical application it can become economical to use waste frying oils from the food industry for PHB production. While waste frying oil is abundant, the main hurdle is putting collection systems in place to obtain this waste resource. Other uses for waste frying oil, such as biodiesel production stimulate collection but also increase prices. In PHB production waste frying oil can be used without filtration, while for biodiesel production it needs to be filtered.

Using waste frying oil to produce PHB can be both cost-effective and environmentally beneficial.

## Competing interests

The authors declare that they have no competing interests.
